# Plasma neurofilament light chain protein is not increased in forensic psychiatric populations: a pilot study

**DOI:** 10.3389/fpsyt.2023.1176266

**Published:** 2023-05-04

**Authors:** Anja Fernqvist, Eirini Alexiou, Henrik Zetterberg, Katarina Howner, Thomas Nilsson, Peter Andiné

**Affiliations:** ^1^Center for Ethics, Law and Mental Health, Department of Psychiatry and Neurochemistry, Institute of Neuroscience and Physiology, The Sahlgrenska Academy, University of Gothenburg, Gothenburg, Sweden; ^2^Department of Forensic Psychiatry, National Board of Forensic Medicine, Gothenburg, Sweden; ^3^Forensic Psychiatric Clinic, Sahlgrenska University Hospital, Gothenburg, Sweden; ^4^Department of Psychiatry and Neurochemistry, Institute of Neuroscience and Physiology, The Sahlgrenska Academy, University of Gothenburg, Mölndal, Sweden; ^5^Clinical Neurochemistry Laboratory, Sahlgrenska University Hospital, Mölndal, Sweden; ^6^Department of Neurodegenerative Disease, UCL Institute of Neurology, London, United Kingdom; ^7^UK Dementia Research Institute, University College London, London, United Kingdom; ^8^Hong Kong Center for Neurodegenerative Diseases, Clear Water Bay, Hong Kong, Hong Kong SAR, China; ^9^Wisconsin Alzheimer’s Disease Research Center, School of Medicine and Public Health, University of Wisconsin-Madison, Madison, WI, United States; ^10^Department of Clinical Neuroscience, Centre for Psychiatry Research, Karolinska Institutet, Stockholm, Sweden

**Keywords:** forensic psychiatry, psychosis, neural injury, biomarker, neurofilament

## Abstract

**Introduction:**

Neurofilament light chain protein (NfL) is a fluid biomarker of neural injury measurable in cerebrospinal fluid and blood. Patients with different neurodegenerative disorders and mild traumatic brain injury display elevated levels of NfL. However, so far, elevated levels of NfL have not been demonstrated in persons with psychiatric disorders. To our knowledge, the occurrence of NfL in the blood has not previously been studied in persons undergoing forensic psychiatric assessment or persons treated in forensic mental health services. Supposedly, these persons suffer from experiences and conditions with a higher risk of neural injury than other psychiatric patients.

**Methods:**

In this pilot study, we investigated plasma levels of NfL in 20 persons undergoing forensic psychiatric assessment and 20 patients at a forensic psychiatric hospital. NfL values were compared with control groups of healthy individuals matched for age and sex.

**Results:**

The prevalence of increased NfL in both forensic groups was low and did not differ compared with the controls. However, some persons undergoing forensic psychiatric assessment showed slightly elevated values.

**Discussion:**

The slightly elevated values were observed in the group investigated closer in time to the index crime, when elevated NfL levels could be expected to be more prevalent due to acute conditions from the time of the offense. This gives reason to look further into this group.

## Introduction

Patients within forensic psychiatric settings are burdened by mental illness, comorbidities, and risk for relapse in violent behavior, making rehabilitation challenging and causing long hospital stays. Poor brain health of the patients has been proposed as one explaining factor that may be the target for future interventions in forensic psychiatry (1). Brain health is correlated with cognitive function, an essential element for the success of forensic psychiatric rehabilitation (2, 3).

Forensic psychiatric patients may display poor brain health for several reasons. First, they often have a psychotic disorder. Schizophrenia is a psychotic disorder with morphological changes in several brain regions (4) and cognitive dysfunction (5). Brain damage may be even more profound in patients with schizophrenia and aggressive behavior (6). Second, many patients have a comorbid substance use disorder with a potential risk for brain damage and cognitive deficits (7). Third, there is a link between traumatic brain injury and violent behavior (8). Forensic psychiatric patients (9) and young violent offenders in prison (10) have a high prevalence of previous traumatic brain injuries. Fourth, forensic psychiatric patients have profound comorbidities with somatic disorders, such as diabetes, hypertension, and metabolic syndrome, factors known to influence the level of NfL (11), which probably contribute to the reduction in estimated life expectancy in these patients (12).

Neurofilament light chain protein (NfL) derives from the neuronal cytoskeleton and is increased in cerebrospinal fluid in several neurodegenerative conditions (13). The levels of NfL in the cerebrospinal fluid correlate with serum and plasma levels. In recent years blood NfL has been established as a biomarker for neuronal injury (11), such as traumatic brain injury (14). The Single molecule array (Simoa) technology has enabled the detection and quantification of clinically useful levels of NfL in the blood (15). In Sweden, plasma NfL was recently introduced as a routine clinical chemistry test (16). Severe mental illness *per se* does not seem to be associated with increased levels of NfL (17–21), although one study found contradicting results in a group of patients with schizophrenia (22). Forensic psychiatric patients may display increased blood NfL levels due to brain health-related comorbidities. However, to our knowledge, there is no study on blood levels of NfL in forensic psychiatric patients.

The present study aimed to investigate the plasma levels of NfL in two forensic psychiatric groups, one undergoing a forensic psychiatric assessment and one in treatment in a forensic psychiatric hospital. We used two clinical forensic psychiatric cohorts being heterogenic regarding psychiatric diagnoses and comorbidities, based on a need for pragmatically designed studies (23) within forensic psychiatric research (24).

## Materials and methods

### Forensic psychiatry in Sweden

The Swedish legal system does not allow criminal defendants to be less accountable or plead not guilty due to legal insanity. Instead, offenders with severe mental disorders can be sentenced to involuntary psychiatric care (25). A forensic psychiatric assessment, performed by the National Board of Forensic Medicine in Sweden, is essential to the criminal court’s decision basis when deciding the sentence. Forensic psychiatric assessments are done at the request of the court. Approximately half of those assessed are judged to have a severe mental disorder. Participants in this study’s forensic psychiatric assessment group had been found guilty of a serious crime, were detained, and underwent psychiatric assessment before the final verdict. The forensic mental health services group participants had all been sentenced to involuntary forensic psychiatric care. Thus, the two groups represented two different time points after the crime.

### Forensic psychiatric assessment group

Participants in the forensic psychiatric assessment group, labeled the assessment group, were recruited from an ongoing study at the National Board of Forensic Medicine in Sweden: “The association between mental disorder, type of crime and relation to the victim: a consecutive study of mentally violent offenders undergoing a forensic psychiatric assessment (RPU 2.0).” In short, the RPU 2.0 compiles data from a psychiatric, psychological, sociological, and nursing care perspective. RPU 2.0 covers persons admitted for forensic psychiatric assessment, aged 18 or more, prosecuted for a violent crime according to the Swedish penal code. Exclusion criteria were insufficient knowledge of the Swedish language to participate without the assistance of an interpreter, inability to give informed consent on participation in research, and ongoing aggressive and threatening behavior. Those meeting the inclusion criteria and considered by the forensic psychiatrist in charge of the assessment capable of giving informed consent were given oral and written information about the RPU 2.0. Beginning in spring 2022, those who consented to participate in RPU 2.0 at the Division of Forensic Psychiatry in Gothenburg were also asked to participate in the sub-study CARERPU 2.0, which adds blood samples, a self-assessment form and body measures to investigate metabolic health and signs of acquired brain damage. All participants provided written informed consent to RPU 2.0 and CARERPU 2.0, respectively. As a part of CARERPU 2.0, this study reports data from the first 20 consecutively included participants to match the size of the forensic mental health services group (see below). Inclusion was performed for 7 months in 2022. During that time, an additional 26 persons were asked to participate but declined. For plasma NfL levels, blood samples were collected in 5 ml EDTA tubes and sent the same day to the Laboratory of Clinical Chemistry at Östra Sahlgrenska University Hospital (SU) of Gothenburg, Sweden, to be analyzed. Items in the RPU 2.0 protocol were used to define selected baseline characteristics; the crime/crimes that led to the executed sentence (index crime) and psychiatric and somatic medical history. Per the diagnostical manuals of choice in Sweden at the time of the study, psychiatric diagnoses were classified in groups as described in the 5th edition of the Diagnostic and Statistical Manual of Mental disorders (DSM-5) (26), while somatic diagnoses were classified in groups according to the 10th International Classification of Diseases (ICD-10) (27). It was noted whether the participant had suffered from a first-time psychotic episode at the time of the index crime and whether he/she had a known history of previous head trauma. Further, it was registered if the participant was prescribed antipsychotic medication or not and whether it constituted single-or multiple treatments.

### Forensic mental health services group

The forensic mental health services group, also called the hospital group, was selected within a retrospective cross-sectional study on clinical medical records (CROSSFOR22) conducted at the Forensic Psychiatric Clinic Rågården at Sahlgrenska University Hospital, Gothenburg. Before data collection, an opt-out procedure suggested by the Swedish Ethical Review Authority was implemented. Information about the study CROSSFOR22 was posted for 2 weeks on bulletin boards in each of the hospital’s four wards for forensic psychiatric rehabilitation. No patient opposed inclusion, and cross-section data were obtained on all 68 patients enrolled at a specific date in 2022. The data collection was conducted in three main steps. First, data from clinical medical records were collected in CROSSFOR22 study protocols by a research nurse with extensive clinical experience. Secondly, an experienced senior consultant and doctoral student evaluated and completed the protocols for psychiatric and somatic medical history. In the third step, an experienced senior consultant and researcher reviewed unclear protocol items (mainly on contradictory and incomplete diagnostical information). Plasma NfL values were found to have been prescribed by the attending physician in 20 patients between September 2020 and January 2022. These 20 patients were included in the study. Due to the strong relation of age to NfL values, age at NfL-sampling rather than at the time of cross-section was recorded (28, 29). Selected baseline characteristics corresponding to the forensic psychiatric assessment group were collected from the CROSSFOR22 protocol. Index crimes were classified as violent or non-violent per the Swedish penal code (30).

### Control groups

For each case, we selected two age-and sex-matched individuals who had no history or clinical symptoms or signs of neurologic disorder from a large study that was conducted to generate normal reference limits for plasma NfL in our laboratory, as previously described in detail (16).

### NfL analysis

Plasma NfL concentration was measured by Simoa using the NF-Light Advantage kit on an HD-X Analyzer according to instructions from the manufacturer (Quanterix, Billerica, MA), as previously described in detail (16). The measurements were performed in clinical laboratory practice by board-certified laboratory technicians blinded to clinical data. Longitudinal stability in the measurements is monitored and ascertained by high and low internal control samples; intra-and inter-assay coefficients of variation were below 10%, and no longitudinal drift was observed.

### Statistics

Statistical analyses were performed in IBM SPSS version 29.0.0. Mann–Whitney U tests were also performed in GraphPad Prism version 9.5.0 to visualize the results in scatter plots. Plasma NfL concentration and age were treated as continuous variables, and other baseline characteristics as categorical, nominal variables. In the hospital group, the length of forensic psychiatric care in months, from the date of admission until the blood sample for NfL analysis, was collected and calculated in SPSS for each participant. After de-identification, the corresponding variables for each study group were registered in separate SPSS data sheets. Baseline characteristics were summarized using SPSS Frequencies. Data were assumed not to be normally distributed. The mean rank values of plasma NfL were calculated with the Mann–Whitney U test to compare the two study groups’ primary outcome (plasma NfL concentration) to the plasma NfL concentrations of the corresponding control groups. The level of significance was set to 0.050.

### Ethics statement

Separate ethical approvals were obtained from the Swedish Ethical Review Authority for RPU2.0 (1016-16), CARERPU2.0 (2021-02420, 2021-05631-02), and CROSSFOR22 (2021-05668-01), respectively.

## Results

### Study group characteristics

Selected baseline characteristics are presented in Table 1. In contrast to the hospital group, the assessment group consisted of both females (30%) and males. The median age was slightly higher than in the hospital group due to a few older participants, as indicated by the interquartile ranges. A primary diagnosis of any psychotic disorder and bipolar disorder, as well as antipsychotic treatment at the time of plasma NfL analysis, were more common in the hospital group (75%) compared with the assessment group (25%). The remaining primary psychiatric diagnoses in the hospital group were few and constituted by neurodevelopmental disorders: (*n* = 3, 15%), personality disorder; (*n* = 1, 5%), and substance-related disorder; (*n* = 1, 5%). In the assessment group, substance-related and addictive disorders were more frequent; (*n* = 6, 30%), as were personality disorders; (*n* = 4, 20%). Three participants were assigned a neurodevelopmental disorder and one a disruptive disorder, impulse-control disorder. As seen in Table 1, somatic diagnoses, including those of metabolic and cardiovascular significance, were more common in the hospital group, as was a history of brain damage. No acute or recent brain damage was reported in either group. Other somatic diagnoses varied greatly in both groups without any significant pattern. The median length of stay at forensic mental health services at the time of blood sample collection was 20.5 months. However, a few participants had a considerably shorter time in forensic psychiatric treatment. There was no participant with a shorter length of stay than 3 months.

**Table 1:** Selected baseline characteristics of participants.

	Forensic psychiatric assessment group (*n* = 20)	Forensic mental health services group (*n* = 20)
Male sex, *n* (%)	14 (70)	20 (100)
Median age (IQR)	35.5 (26.5–48.0)	29.5 (25.0–32.8)
Any psychosis/bipolar I, *n* (%)	5 (25)	15 (75)
Any substance use disorder, *n* (%)	11 (55)	13 (65)
Alcohol-related disorders, *n* (%)	4 (20)	1 (5)
Cannabis-related disorders, *n* (%)	3 (15)	4 (20)
Opioid-related disorders, *n* (%)	5 (25)	0 (0)
Sedative-, Hypnotic-, or Anxiolytic-related disorders, *n* (%)	2 (10)	0 (0)
Stimulant-related disorders, *n* (%)	5 (25)	1 (5)
Other (or unknown) substance-related disorders, *n* (%)	1 (0)	0 (0)
Multiple drug use*, *n* (%)	0 (0)	10 (50)
Any somatic diagnosis, *n* (%)	8 (40)	11 (55)
Any neurological disorder, *n* (%)	1 (5)	0 (0)
Any endocrine, nutritional and metabolic diseases, *n* (%)	3 (15)	6 (30)
Any disease of the circulatory system, *n* (%)	0	1 (5)
Any history of brain damage, *n* (%)	2 (10)	5 (25)
Antipsychotic treatment at NfL analysis, *n* (%)	6 (30)	17 (85)
Length of stay in months at forensic hospital at NfL analysis, median (IQR)	**	20.5 (3–27)

NfL data recorded from 2020 through 2022.

NfL, Neurofilament light chain protein; *n*, number of participants; IQR, interquartile range; *, According to ICD-10; **, not applicable. Participants may have several specific diagnoses.

### Plasma NfL levels

**Figure 1:**
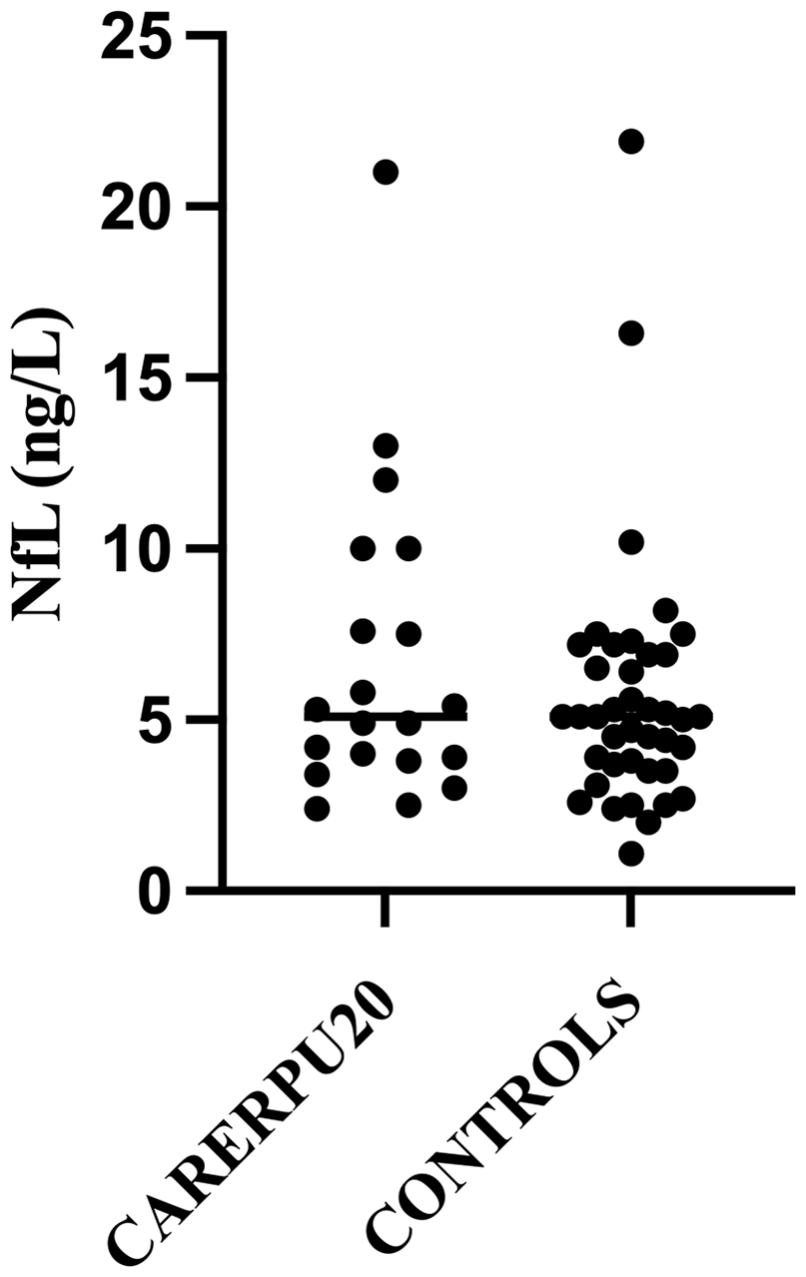
Forensic psychiatric assessment group.

**Figure 2:**
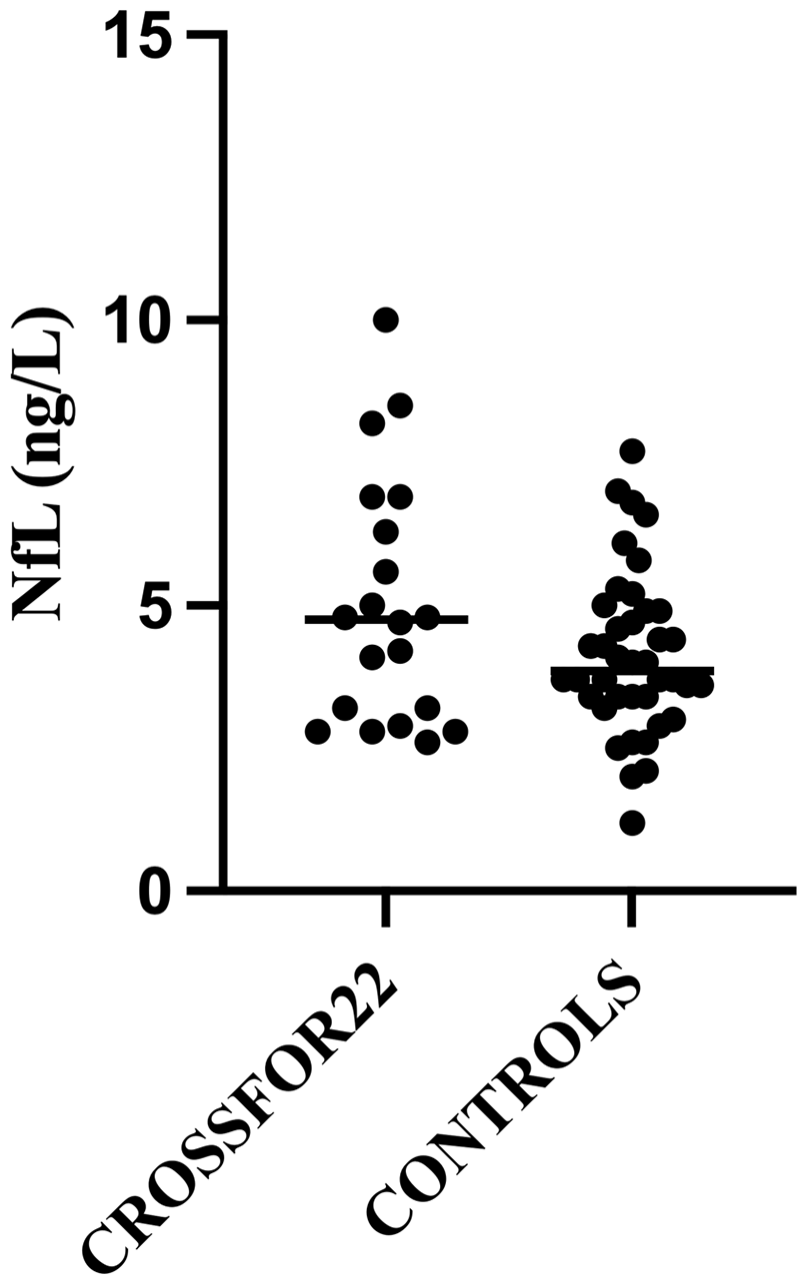
Forensic mental health services group.

No significant differences between the study groups and the controls were detected (Figures 1, 2 and Table 2). When examining individual NfL concentrations, we found that four participants in the assessment group had slightly elevated concentrations compared with age-adjusted reference values, while this was true for none of the participants in the hospital group. The age-adjusted clinical reference limit for all deviating participants was <10 nanograms per liter (ng/L) (16), and NfL values of the four deviating participants were 10, 10, 13, and 21 ng/l, respectively. Out of these four persons, two had an alcohol-related disorder, one had an opioid-related disorder and a hypothyroid disorder, and one had diabetes. The NfL value 12 ng/l seen in Figure 1 represents a participant of higher age where the age-adjusted reference value is <35 ng/l (16).

**Table 2:** NfL values, descriptive statistics and significance levels of comparative analyses.

	Mean Nfl value, ng/L (CI)	Median NfL value, ng/L (IQR)	Value of *p*, comparative analysis of mean ranks in Mann Whitney U test
Forensic psychiatric assessment group (*n* = 20)	6.7 (4.6–8.9)	5.1 (3.8–9.4)	0.471 (n.s.)
Controls of Forensic psychiatric assessment group (*n* = 40)	5.7 (4.7–6.8)	5.1 (3.6–6.9)	–
Forensic mental health services group (*n* = 20)	5.0 (4.0–6.0)	4.8 (3.0–6.8)	0.233 (n.s.)
Controls of Forensic mental health services group (*n* = 40)	4.1 (3.7–4.6)	3.9 (3.4–4.9)	–

NfL, Neurofilament light chain protein; *n*, number of participants; IQR, interquartile range; n.s., not significant.

## Discussion

In this pilot study, we investigated the blood levels of NfL in two groups of forensic subjects. The two groups represented two different time points after the crime. The assessment group was investigated weeks-months after the crime and prior to an expected verdict, a period characterized by acute psychiatric morbidity, recent substance use and stress. The hospital group was investigated months-years after the crime, with a median time of forensic psychiatric care and treatment of 20.5 months. This group was possibly to a greater extent stabilized, considering psychiatric morbidity, recent substance use and stress compared to the assessment group. The two forensic psychiatric groups’ mean rank of blood levels did not differ compared with matched controls. However, some participants from the assessment group showed slightly elevated blood values of NfL compared with clinically used, age-adjusted reference values, which gives reason to look further into this group.

There may be several reasons for the lack of increased blood levels of NfL in the two forensic psychiatric groups. We will mainly discuss the findings in relation to neurological disorders, psychiatric disorders, and substance use disorders. First, NfL is a cytoskeletal protein specific for neurons, which is released to the cerebrospinal fluid and blood in situations of neuronal damage (11, 13). Increased blood NfL has been reported in neurological conditions with a distinct temporal nature, such as stroke, multiple sclerosis, and traumatic brain injury, but also in more chronic conditions, such as Alzheimer’s disease and parkinsonian disorders (11, 13). These conditions were absent in the two forensic psychiatric groups of patients except for previous traumatic brain injury. Studies have reported an association between violent crime and traumatic brain injury (8, 10). In the forensic psychiatric assessment group, 10% reported previous head trauma, while in the forensic psychiatric hospital group, the corresponding figure was 25%. However, no recent or acute head trauma was reported in either group.

Second, Swedish forensic psychiatric patients all have a severe mental disorder, with about two-thirds having a psychotic disorder, mainly schizophrenia (31). In the present study, the prevalence of schizophrenia spectrum and other psychotic disorders was 20 and 75% in the assessment and hospital groups, respectively. There is growing evidence that primary psychiatric disorders, in general, and psychotic disorders, in particular, are not characterized by increased levels of NfL in the cerebrospinal fluid (18, 20) or blood (16–18), not even in patients with treatment-resistant schizophrenia (21), a group with profound cognitive deficits (32) and possibly the group of schizophrenia patients with most neuronal damage (33). One study has shown significantly increased plasma NfL in schizophrenia patients compared with controls, although the absolute difference was small (22). The currently unknown neurodegenerative processes in psychotic disorders may be without NfL increase, or they may occur at specific time points of the disorder [see (21) for discussion] not detected in the present study. Considering these findings, it was not surprising that we did not find elevated blood NfL levels in forensic psychiatric patients.

Third, a growing number of studies also show increased blood NfL levels in persons with different substance use disorders. Alcohol use disorder is associated with increased blood NfL (34), and there seem to be relationships between the dose of alcohol use, blood levels of NfL, and gray matter damage in the brain (35). Blood levels of NfL have even been shown to correlate to the treatment effect of transcranial magnetic stimulation on alcohol use disorder (36). In addition, other drugs than alcohol have also been shown to affect NfL. Users of ketamine (37) and cocaine (38), psychoactive drugs associated with cognitive deficits and brain structure alterations, may have increased blood levels of NfL. In contrast, the use of ecstasy does not seem to affect NfL (39). In the present study, a substance use disorder diagnosis was present in 50 and 65% of the patients in the assessment and hospital groups, respectively. In the assessment group, active substance use is likely to have occurred more recently than in the hospital group. This might explain the findings of a few slightly elevated NfL levels in the assessment group compared with clinically applied reference values. In fact, three out of four persons with elevated NfL levels did have a substance use disorder.

Finally, some additional comments on the finding that a few subjects from the assessment group displayed slightly elevated blood NfL levels. A forensic psychiatric assessment most likely occurs within weeks or months after the index crime in offenders with a suspected severe mental disorder. Thus, acute conditions from the time of the offense, possibly associated with neuronal damage and NfL leakage, would be closer in time to the forensic psychiatric assessment than patients undergoing forensic psychiatric care. NfL dynamics in CSF and blood after acute brain injury mirrors each other with slow increases days to weeks after the injury, reaching peak concentrations after around 2–3 months, followed by reduced concentrations with an apparent half-life of 2–3 months (40). Elevated blood NfL levels could therefore be expected to be more prevalent during the forensic psychiatric assessment. In addition, patients with 3 months of alcohol withdrawal still have elevated blood NfL levels, although not as prominent as during the first day of alcohol abstinence (41). Another acute condition with elevated blood NfL levels is anti-NMDA receptor encephalitis (42). Speculatively, some of the patients with a severe mental disorder due to a first episode of psychosis may, in fact, have a so-called autoimmune psychosis (43) with NMDA receptor antibodies (44). Other factors that may cause elevated blood NfL levels are higher age and risk factors for cardiovascular disorders such as high blood pressure, diabetes, and smoking (11). The present study’s median age was 35.5 and 29.5 years in the two groups, respectively. Patients undergoing forensic psychiatric assessment had few somatic diagnoses related to metabolic and cardiovascular risk factors. About half of the patients at the forensic psychiatric hospital had a somatic diagnosis, with 30% having a diagnosis related to metabolic and cardiovascular disorders. There are also some conditions that may lower the blood levels of NfL (11). These include conditions with increased blood volume. Such a condition relevant to almost half of the forensic psychiatric patients is a high body mass index (45), which may lower the group mean of blood NfL in forensic psychiatric groups compared with controls. Unfortunately, in our study, we could not match controls regarding body mass index. Another condition speculatively lowering the blood levels of NfL is in a review by Barro and coworkers referred to as disease-modifying treatment (11). This would represent all treatments putatively targeting neurodegenerative processes given to patients from the arrest to the time of NfL testing. For forensic psychiatric patients, this would include antipsychotic and other psychopharmacological treatment but also non-pharmacological factors such as abstinence from alcohol and other drugs and a routine-based low stimuli environment.

### Strengths and limitations

We consider the choice of groups a strength of our study since they are challenging to include in medical research, and previous studies are lacking. The participants are psychiatrically well characterized due to the forensic psychiatric assessment. Another strength is that the two groups were included at different times in the forensic psychiatric timeline. Limitations are the small number of participants in each group. This makes it difficult to detect any diagnostic-specific changes in NfL. In addition, the most aggressive and severely psychiatrically ill were excluded, as well as potential participants with scarce language skills. Another limitation of the study is the lack of imaging data on any structural brain damage.

## Data availability statement

The original contributions presented in the study are included in the article/Supplementary material, further inquiries can be directed to the corresponding author.

## Ethics statement

The studies involving human participants were reviewed and approved by the Swedish Ethical Review Authority. The patients/participants in CARERPU 2.0 provided their written informed consent to participate in this study. The patients/participants in CROSSFOR22 were informed about the study and had the option to oppose inclusion.

## Author contributions

AF collected the data. AF and EA organized the database and performed the statistical analyses. AF analyzed the results supervised by PA. AF and PA wrote the first draft of the manuscript. HZ, KH, TN, and EA participated the manuscript revisions. AF, PA, HZ, TN, and KH contributed to the design of the study and conclusion. All authors finally read and approved the submitted version of the manuscript.

## Funding

The work was supported by the Swedish Research Council for Health, Working Life and Welfare (#2018-01409), the Dahrén Foundations, and the Foundation for Rehabilitation and Medical Research. HZ is a Wallenberg Scholar supported by grants from the Swedish Research Council (#2022-01018), the European Union’s Horizon Europe research and innovation programme under grant agreement No. 101053962, Swedish State Support for Clinical Research (#ALFGBG-71320), the Alzheimer Drug Discovery Foundation (ADDF), United States (#201809-2016862), the AD Strategic Fund and the Alzheimer’s Association (#ADSF-21-831376-C, #ADSF-21-831381-C, and #ADSF-21-831377-C), the Bluefield Project, the Olav Thon Foundation, the Erling-Persson Family Foundation, Stiftelsen för Gamla Tjänarinnor, Hjärnfonden, Sweden (#FO2022-0270), the European Union’s Horizon 2020 research and innovation programme under the Marie Skłodowska-Curie grant agreement No. 860197 (MIRIADE), the European Union Joint Programme – Neurodegenerative Disease Research (JPND2021-00694), and the UK Dementia Research Institute at UCL (UKDRI-1003).

## Conflict of interest

HZ has served at scientific advisory boards and/or as a consultant for Abbvie, Acumen, Alector, Alzinova, ALZPath, Annexon, Apellis, Artery Therapeutics, AZTherapies, CogRx, Denali, Eisai, Nervgen, Novo Nordisk, Optoceutics, Passage Bio, Pinteon Therapeutics, Prothena, Red Abbey Labs, reMYND, Roche, Samumed, Siemens Healthineers, Triplet Therapeutics, and Wave, has given lectures in symposia sponsored by Cellectricon, Fujirebio, Alzecure, Biogen, and Roche, and is a co-founder of Brain Biomarker Solutions in Gothenburg AB (BBS), which is a part of the GU Ventures Incubator Program (outside submitted work).

The remaining authors declare that the research was conducted in the absence of any commercial or financial relationships that could be construed as a potential conflict of interest.

## Publisher’s note

All claims expressed in this article are solely those of the authors and do not necessarily represent those of their affiliated organizations, or those of the publisher, the editors and the reviewers. Any product that may be evaluated in this article, or claim that may be made by its manufacturer, is not guaranteed or endorsed by the publisher.

## References

[1] Andiné P Bergman H. Focus on brain health to improve care, treatment and rehabilitation in forensic psychiatry. Front Psych. (2019) 10:840. doi: 10.3389/fpsyt.2019.00840, PMID: 31849721 PMC6901922

[2] O’Reilly K Donohoe G O’Sullivan D Coyle C Corvin A O’Flynn P. A randomized controlled trial of cognitive remediation for a national cohort of forensic patients with schizophrenia or schizoaffective disorder. BMC Psychiatry. (2019) 19:27. doi: 10.1186/s12888-019-2018-6, PMID: 30646884 PMC6334394

[3] Puzzo I Sedgwick O Kelly R Greer B Kumari V Guðjónsson G. Attention problems predict risk of violence and rehabilitative engagement in mentally disordered offenders. Front Psych. (2019) 10:279. doi: 10.3389/fpsyt.2019.00279, PMID: 31133891 PMC6514136

[4] Howes OD Cummings C Chapman GE Shatalina E. Neuroimaging in schizophrenia: an overview of findings and their implications for synaptic changes. Neuropsychopharmacol. (2023) 48:151–67. doi: 10.1038/s41386-022-01426-x, PMID: 36056106 PMC9700830

[5] McCleery A Nuechterlein KH. Cognitive impairment in psychotic illness: prevalence, profile of impairment, developmental course, and treatment considerations. Dialogues Clin Neurosci. (2019) 21:239–48. doi: 10.31887/DCNS.2019.21.3/amccleery, PMID: 31749648 PMC6829172

[6] Fjellvang M Grøning L Haukvik UK. Imaging violence in schizophrenia: a systematic review and critical discussion of the MRI literature. Front Psych. (2018) 9:333. doi: 10.3389/fpsyt.2018.00333, PMID: 30083111 PMC6064955

[7] Unterrainer HF Hiebler-Ragger M Koschutnig K Fuchshuber J Ragger K Perchtold CM. Brain structure alterations in poly-drug use: reduced cortical thickness and white matter impairments in regions associated with affective, cognitive, and motor functions. Front Psych. (2019) 10:667. doi: 10.3389/fpsyt.2019.00667, PMID: 31616326 PMC6763614

[8] Fazel S Lichtenstein P Grann M Långström N. Risk of violent crime in individuals with epilepsy and traumatic brain injury: a 35-year Swedish population study. PLoS Med. (2011) 8:e1001150. doi: 10.1371/journal.pmed.1001150, PMID: 22215988 PMC3246446

[9] Colantonio A Stamenova V Abramowitz C Clarke D Christensen B. Brain injury in a forensic psychiatry population. Brain Inj. (2007) 21:1353–60. doi: 10.1080/02699050701785054, PMID: 18066937

[10] Katzin S Andiné P Hofvander B Billstedt E Wallinius M. Exploring traumatic brain injuries and aggressive antisocial behaviors in Young male violent offenders. Front Psych. (2020) 11:507196. doi: 10.3389/fpsyt.2020.507196, PMID: 33192641 PMC7581682

[11] Barro C Chitnis T Weiner HL. Blood neurofilament light: a critical review of its application to neurologic disease. Ann Clin Transl Neurol. (2020) 7:2508–23. doi: 10.1002/acn3.51234, PMID: 33146954 PMC7732243

[12] Ojansuu I Putkonen H Tiihonen J. Mortality among forensic psychiatric patients in Finland. Nord J Psychiatry. (2015) 69:25–7. doi: 10.3109/08039488.2014.908949 24802122

[13] Khalil M Teunissen CE Otto M Piehl F Sormani MP Gattringer T. Neurofilaments as biomarkers in neurological disorders. Nat Rev Neurol. (2018) 14:577–89. doi: 10.1038/s41582-018-0058-z 30171200

[14] Shahim P Zetterberg H. Neurochemical markers of traumatic brain injury: relevance to acute diagnostics, disease monitoring, and neuropsychiatric outcome prediction. Biol Psychiatry. (2022) 91:405–12. doi: 10.1016/j.biopsych.2021.10.010, PMID: 34857362

[15] Gisslén M Price RW Andreasson U Norgren N Nilsson S Hagberg L. Plasma concentration of the Neurofilament light protein (NFL) is a biomarker of CNS injury in HIV infection: a cross-sectional study. EBioMedicine. (2016) 3:135–40. doi: 10.1016/j.ebiom.2015.11.036, PMID: 26870824 PMC4739412

[16] Simrén J Andreasson U Gobom J Suarez Calvet M Borroni B Gillberg C. Establishment of reference values for plasma neurofilament light based on healthy individuals aged 5–90 years. Brain Commun. (2022) 4:fcac174. doi: 10.1093/braincomms/fcac174, PMID: 35865350 PMC9297091

[17] Al Shweiki MR Steinacker P Oeckl P Hengerer B Danek A Fassbender K. Neurofilament light chain as a blood biomarker to differentiate psychiatric disorders from behavioural variant frontotemporal dementia. J Psychiatr Res. (2019) 113:137–40. doi: 10.1016/j.jpsychires.2019.03.019, PMID: 30953863

[18] Ducharme S Dols A Laforce R Devenney E Kumfor F van den Stock J. Recommendations to distinguish behavioural variant frontotemporal dementia from psychiatric disorders. Brain. (2020) 143:1632–50. doi: 10.1093/brain/awaa018, PMID: 32129844 PMC7849953

[19] Katisko K Cajanus A Jääskeläinen O Kontkanen A Hartikainen P Korhonen VE. Serum neurofilament light chain is a discriminative biomarker between frontotemporal lobar degeneration and primary psychiatric disorders. J Neurol. (2020) 267:162–7. doi: 10.1007/s00415-019-09567-8, PMID: 31595378 PMC6954884

[20] Eratne D Janelidze S Malpas CB Loi S Walterfang M Merritt A. Plasma neurofilament light chain protein is not increased in treatment-resistant schizophrenia and first-degree relatives. Aust N Z J Psychiatry. (2022) 56:1295–305. doi: 10.1177/00048674211058684 35179048

[21] Eratne D Loi SM Li QX Stehmann C Malpas CB Santillo A. Cerebrospinal fluid neurofilament light chain differentiates primary psychiatric disorders from rapidly progressive, Alzheimer's disease and frontotemporal disorders in clinical settings. Alzheimers Dement. (2022) 18:2218–33. doi: 10.1002/alz.12549, PMID: 35102694

[22] Rodrigues-Amorim D Rivera-Baltanás T del Carmen V-CM Rodriguez-Jamardo C de Las Heras E Barreiro-Villar C. Plasma β-III tubulin, neurofilament light chain and glial fibrillary acidic protein are associated with neurodegeneration and progression in schizophrenia. Sci Rep. (2020) 10:14271. doi: 10.1038/s41598-020-71060-4 32868793 PMC7459108

[23] Paulus MP. Evidence-based pragmatic psychiatry-a call to action. JAMA Psychiat. (2017) 74:1185–6. doi: 10.1001/jamapsychiatry.2017.2439, PMID: 29094142

[24] Howner K Andiné P Engberg G Ekström EH Lindström E Nilsson M. Pharmacological treatment in forensic psychiatry-a systematic review. Front Psych. (2019) 10:963. doi: 10.3389/fpsyt.2019.00963 PMC6976536 32009993

[25] Radovic S Meynen G Bennet T. Introducing a standard of legal insanity: the case of Sweden compared to the Netherlands. Int J Law Psychiatry. (2015) 40:43–9. doi: 10.1016/j.ijlp.2015.04.009 26003234

[26] American Psychiatric Association. Diagnostic and statistical manual of mental disorders DSM-5. 5th ed. Arlington, VA: American Psychiatric Association (2013).

[27] World Health Organization. International statistical classification of diseases and related health problems. 11th ed. Genève, Switzerland: World Health Organization, ICD-10 (2019).

[28] Khalil M Pirpamer L Hofer E Voortman MM Barro C Leppert D. Serum neurofilament light levels in normal aging and their association with morphologic brain changes. Nat Commun. (2020) 11:812. doi: 10.1038/s41467-020-14612-6, PMID: 32041951 PMC7010701

[29] Gaetani L Blennow K Calabresi P Di Filippo M Parnetti L Zetterberg H. Neurofilament light chain as a biomarker in neurological disorders. J Neurol Neurosurg Psychiatry. (2019) 90:870–81. doi: 10.1136/jnnp-2018-320106, PMID: 30967444

[30] Sveriges regering. SFS 1962: 700 Brottsbalk. Stockholm: Sveriges regering (1962). 621221 p.

[31] Degl' Innocenti A Alexiou E Andiné P Striskaite J Nilsson T. A register-based comparison study of Swedish patients in forensic psychiatric care 2010 and 2018. Int J Law Psychiatry. (2021) 77:101715. doi: 10.1016/j.ijlp.2021.101715, PMID: 34052684

[32] Iasevoli F Avagliano C Altavilla B Barone A D'Ambrosio L Matrone M. Disease severity in treatment resistant schizophrenia patients is mainly affected by negative symptoms, which mediate the effects of cognitive dysfunctions and neurological soft signs. Front Psych. (2018) 9:553. doi: 10.3389/fpsyt.2018.00553, PMID: 30429802 PMC6220073

[33] Mouchlianitis E McCutcheon R Howes OD. Brain-imaging studies of treatment-resistant schizophrenia: a systematic review. Lancet Psychiatry. (2016) 3:451–63. doi: 10.1016/S2215-0366(15)00540-4, PMID: 26948188 PMC5796640

[34] Li Y Duan R Gong Z Jing L Zhang T Zhang Y. Neurofilament light chain is a promising biomarker in alcohol dependence. Front Psych. (2021) 12:754969. doi: 10.3389/fpsyt.2021.754969, PMID: 34867542 PMC8637455

[35] Karoly HC Skrzynski CJ Moe EN Bryan AD Hutchison KE. Exploring relationships between alcohol consumption, inflammation, and brain structure in a heavy drinking sample. Alcohol Clin Exp Res. (2021) 45:2256–70. doi: 10.1111/acer.14712, PMID: 34523725 PMC8642310

[36] Zhang T Song B Li Y Duan R Gong Z Jing L. Neurofilament light chain as a biomarker for monitoring the efficacy of transcranial magnetic stimulation on alcohol use disorder. Front Behav Neurosci. (2022) 16:831901. doi: 10.3389/fnbeh.2022.831901, PMID: 35197833 PMC8859255

[37] Liu YL Bavato F Chung AN Liu TH Chen YL Huang MC. Neurofilament light chain as novel blood biomarker of disturbed neuroaxonal integrity in patients with ketamine dependence. World J Biol Psychiatry. (2021) 22:713–21. doi: 10.1080/15622975.2021.1907709, PMID: 33783299

[38] Bavato F Kexel AK Kluwe-Schiavon B Maceski A Baumgartner MR Seifritz E. A longitudinal investigation of blood neurofilament light chain levels in chronic cocaine users. Mol Neurobiol. (2023):1–10. doi: 10.1007/s12035-023-03327-6 PMC10224834 37000398

[39] Zimmermann J Friedli N Bavato F Stämpfli P Coray R Baumgartner MR. White matter alterations in chronic MDMA use: evidence from diffusion tensor imaging and neurofilament light chain blood levels. Neuroimage Clin. (2022) 36:103191. doi: 10.1016/j.nicl.2022.103191, PMID: 36126513 PMC9486575

[40] Bergman J Dring A Zetterberg H Blennow K Norgren N Gilthorpe J. Neurofilament light in CSF and serum is a sensitive marker for axonal white matter injury in MS. Neurol Neuroimmunol Neuroinflamm. (2016) 3:e271. doi: 10.1212/NXI.0000000000000271, PMID: 27536708 PMC4972001

[41] Clergue-Duval V Vrillon A Jeanblanc J Questel F Azuar J Fouquet G. Plasma tau, NfL, GFAP and UCHL1 as candidate biomarkers of alcohol withdrawal-associated brain damage: a pilot study. Addict Biol. (2022) 27:e13232. doi: 10.1111/adb.13232, PMID: 36301211

[42] Guasp M Martín-Aguilar L Sabater L Bioque M Armangué T Martínez-Hernández E. Neurofilament light chain levels in anti-NMDAR encephalitis and primary Psychiatric psychosis. Neurology. (2022) 98:e1489–98. doi: 10.1212/WNL.0000000000200021, PMID: 35145006

[43] Pollak TA Lennox BR Müller S Benros ME Prüss H Tebartz van Elst L. Autoimmune psychosis: an international consensus on an approach to the diagnosis and management of psychosis of suspected autoimmune origin. Lancet. Psychiatry. (2020) 7:93–108. doi: 10.1016/S2215-0366(19)30290-1, PMID: 31669058

[44] Kelleher E McNamara P Dunne J Fitzmaurice B Heron EA Whitty P. Prevalence of N-methyl-d-aspartate receptor antibody (NMDAR-ab) encephalitis in patients with first episode psychosis and treatment resistant schizophrenia on clozapine, a population based study. Schizophr Res. (2020) 222:455–61. doi: 10.1016/j.schres.2019.11.023, PMID: 32499165

[45] Hilton NZ Ham E Hill S Emmanuel T Konkolÿ TB. Predictors of weight gain and metabolic indexes among men admitted to forensic Psychiatric hospital. Int J Forensic Ment Health. (2022) 21:164–74. doi: 10.1080/14999013.2021.1952356

